# Node-RADS category on preoperative CT predicts prognosis in patients with papillary renal cell carcinoma

**DOI:** 10.1007/s00330-025-11446-y

**Published:** 2025-02-20

**Authors:** Xiaoxia Li, Dengqiang Lin, Ying Xiong, Weifeng Lin, Shaoting Zhang, Shunfa Huang, Jianjun Zhou, Chenchen Dai

**Affiliations:** 1 Department of Radiology, Zhongshan Hospital (Xiamen), Fudan University, Xiamen, China; 2 Department of Urology, Zhongshan Hospital (Xiamen), Fudan University, Xiamen, China; 3 Department of Urology, Zhongshan Hospital, Fudan University, Shanghai, China; 4 Department of Information, Zhongshan Hospital (Xiamen), Fudan University, Xiamen, China; 5 Department of Radiology, Shandong Cancer Hospital and Institute, Shandong First Medical University and Shandong Academy of Medical Sciences, Jinan, China; 6 Department of Medical Imaging, Xiamen Municipal Clinical Research Center for Medical Imaging, Xiamen, China; 7 Department of Medical Imaging, Fujian Province Key Clinical Specialty for Medical Imaging, Xiamen, China; 8 Department of Imaging Big Data and Artificial Intelligence, Xiamen Key Laboratory of Clinical Transformation of Imaging Big Data and Artificial Intelligence, Xiamen, China; 9 Department of Radiology, Zhongshan Hospital, Fudan University, Shanghai, China

**Keywords:** Papillary renal cell carcinoma, Node-RADS, Prognosis, Progression-free survival, Cancer-specific survival

## Abstract

**Objectives:**

This research focused on investigating the relationship between the Node Reporting and Data System (Node-RADS) categories, determined via preoperative CT, and the outcomes of progression-free survival (PFS) and cancer-specific survival (CSS) in individuals diagnosed with papillary renal cell carcinoma (pRCC).

**Methods:**

A retrospective multicenter study initially enrolled 454 patients, with 218 eligible for analysis following partial nephrectomy or radical resection for pRCC. Prognostic factors related to PFS and CSS in pRCC patients were identified through univariate and multivariate Cox regression analyses. Subsequently, the prognostic value of Node-RADS was assessed and compared with the existing pRCC risk stratification model.

**Results:**

In total, 218 patients (mean age, 58 years; men, 164 [75.2%]) with pRCC (186 Node-rads I tumors (85.3%), 10 Node-rads II tumors (4.6%), and 22 Node-rads III tumors (10.1%)) were included. The Node-RADS category emerged as an independent prognostic factor for PFS (III vs II vs I, hazard ratio (HR) 4.250, *p* < 0.001) and CSS (III vs II vs I, HR 4.466; *p* < 0.001). When the Node-RADS category was incorporated into Leibovich’s model, the resulting combined model demonstrated a significant improvement in predictive accuracy (C-index: 0.865 versus 0.755, *p* = 0.005 for PFS; and 0.921 versus 0.835, *p* = 0.01 for CSS).

**Conclusion:**

The Node-RADS category has been identified as a more accurate predictor of prognosis for pRCC, regardless of pathologic lymph node involvement. These findings need further confirmation in prospective studies.

**Key Points:**

***Question*** *Lymph node status is important for papillary renal cell carcinoma prognosis, and there is a lack of consensus on radiological evaluation.*

***Findings*** *Node-RADS is an independent predictor of progression-free survival and cancer-specific survival in papillary renal cell carcinoma.*

***Clinical relevance*** *The Node Reporting and Data System category improves the accuracy of the Leibovich model for prognosis, which can help clinical practitioners select individualized treatment plans for each patient.*

**Graphical Abstract:**

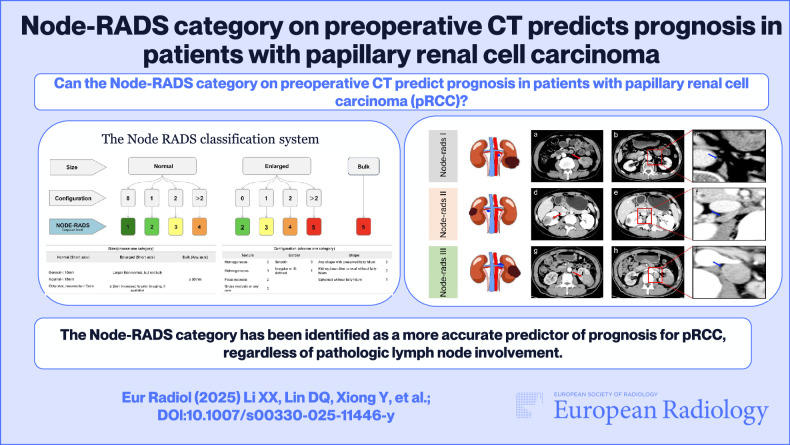

## Introduction

Papillary renal cell carcinoma (pRCC) constitutes 15–20% of kidney cancers, with approximately 10% of cases at risk of progression [1]. Like many solid tumors, this disease is incurable in the metastatic stage and typically carries a poor prognosis [2].

Unlike patients with advanced clear cell RCC, those with advanced pRCC generally derive minimal benefits from targeted treatments [3–6]. Most risk models have primarily focused on clear cell RCC, and well-defined prognostic models for pRCC are lacking. Therefore, identifying a reliable parameter or risk model for patients with pRCC is of paramount importance.

Lymph node involvement (LNI) markedly impacts the prognosis of localized renal cancer [7, 8], particularly in cases of papillary renal cell carcinoma (pRCC) [8]. Accurately determining the significance of pathologic nodal involvement in renal cell carcinoma (RCC) can be challenging and may be underestimated. It is noteworthy that only about 1% of RCC patients are classified as having lymph node involvement (LNI) post-operatively [9, 10]. This phenomenon can be attributed to several factors. First, the decision to perform regional lymph node dissection (LND) during surgery remains debatable [11], and not all patients undergo LND. This introduces an observational bias since only patients selected for LND can be identified as node-positive. Second, among patients who do undergo LND, there is a selection bias due to the lack of standardized guidelines regarding the extent of LND. This variability in practice potentially leads to the missed detection of positive lymph nodes [12].

Image-based assessment of lymph node status is a crucial aspect in evaluating various tumors. Several recent studies have highlighted the prognostic value of clinically positive lymph nodes in RCC [13–16], but there is no universally accepted definition for clinically positive lymph nodes. Many studies define them based on a short diameter greater than 1 cm, however, this criterion may need to be re-evaluated due to the frequent oversight of micrometastases, which hinders widespread clinical utility [17]. Therefore, additional computer-aided procedures for quantitative analysis are necessary.

Recently, the launch of Node-RADS 1.0 in 2021 aimed to standardize the assessment of lymph nodes in cancer, categorizing the suspicion levels of lymph node involvement with detailed criteria [18]. Besides the conventional ‘size’ criterion, Node-RADS incorporates a ‘configuration’ criterion that evaluates morphological features such as ‘texture,’ ‘border,’ and ‘shape’ for each lymph node. Node-RADS has been initially explored in other tumors [19–21], however, the role of Node-RADS in renal cancer prognosis is still unknown.

The purpose of this study was to assess the prognostic significance of Node-RADS for progression-free survival (PFS) and cancer-specific survival (CSS) in patients diagnosed with pRCC.

## Materials and methods

### Patients

**Fig. 1:**
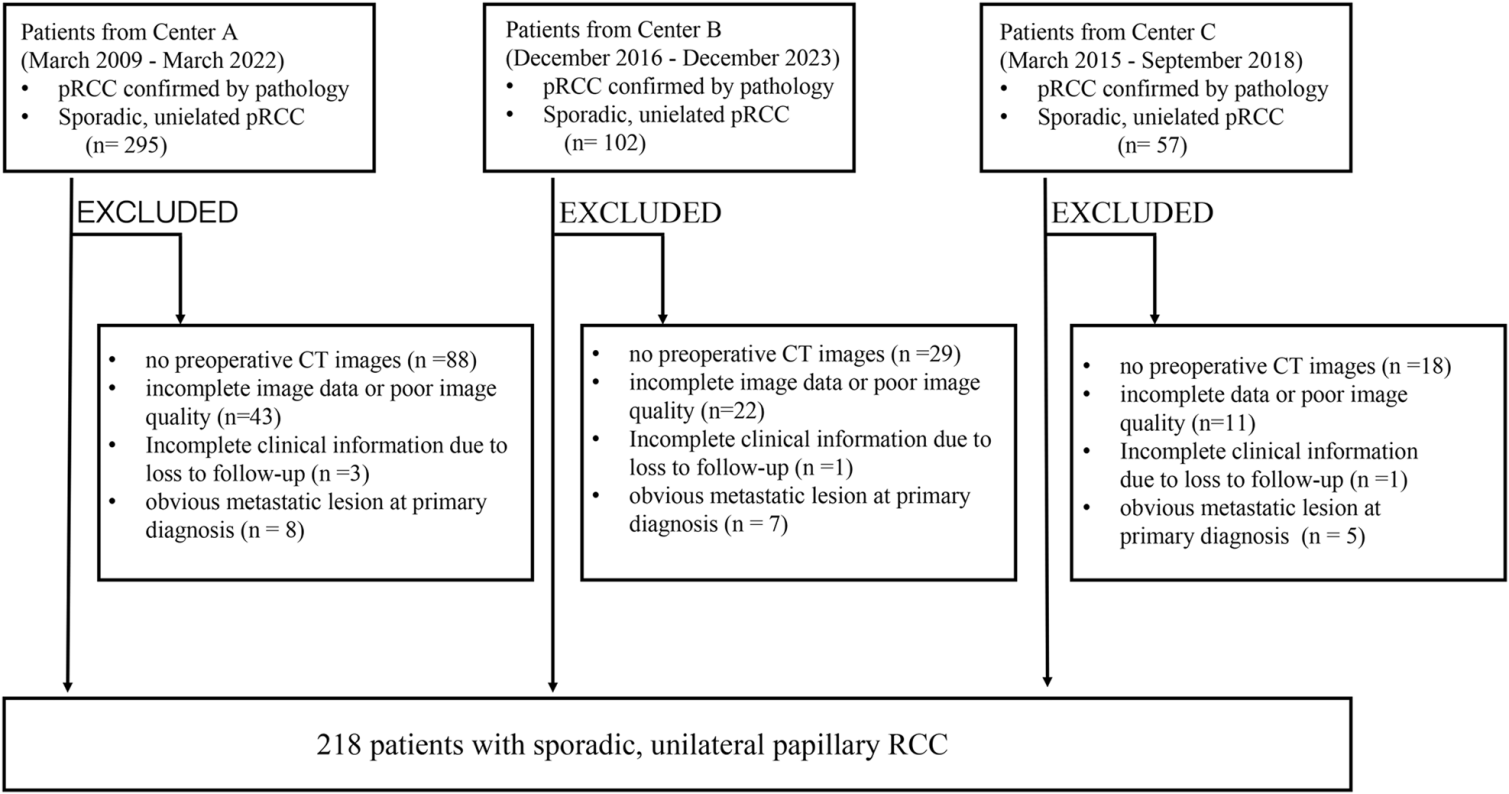
Flow chart of inclusion and exclusion criteria of the study sample

This retrospective study was conducted across multiple centers, following the ethical standards outlined in the Declaration of Helsinki and receiving approval from the ethics committees of the respective participating hospitals. The requirement for informed consent was waived. The study included a total of 218 patients with sporadic, unilateral papillary renal cell carcinoma (pRCC) who underwent either radical or partial nephrectomies between March 2009 and December 2023. The following exclusion criteria were: (1) no preoperative CT image, (2) incomplete image data or poor image quality, (3) incomplete clinical information due to loss to follow-up, and (4) obvious metastatic lesion at primary diagnosis. Inclusion and exclusion criteria are shown in Fig. 1.

### Exposure and outcomes

Clinical data collected for analysis encompassed patient demographics such as age and sex, details of surgical procedures (radical or partial, open or laparoscopic, lymph node dissection or not), adjuvant treatments, the study assessed several factors at the time of diagnosis, including Eastern Cooperative Oncology Group Performance Status (ECOG-PS) and reported symptoms [22]. Pathological characteristics were evaluated by a seasoned urologic pathologist with over 15 years of experience in genitourinary pathology. The features considered included histologic subtype classification (type I, type II, or unclassified) [23], TNM staging according to the eighth edition [24], criteria from the 2016 grading system of the World Health Organization/International Society of Urological Pathology [25], invasion of perinephric or renal sinus fat, presence of venous thrombus, involvement of positive lymph nodes, evidence of tumor necrosis, differentiation with sarcomatoid features, and evaluation of tumor size based on the cross-sectional imaging showing the longest diameter.

Progression was defined as local ipsilateral recurrence, contralateral recurrence, or distant metastasis on the basis of follow-up CT or MR imaging evaluation and clinical records. Death from RCC was defined based on death certificate review or death following a clinical record for metastatic pRCC. Progression-free survival (PFS) was determined by measuring the duration from the date of surgery to the date of tumor progression. Cancer-specific survival (CSS) was calculated from the date of surgery to the date of death from RCC. Progression or death dates were acquired from medical records or phone inquiries by an author who was not involved in the imaging analysis.

### CT and Node‑RADS evaluation

CT scanners were utilized, which included MDCT scanners with either 16 or 64 detector rows. The specific CT parameters can be found in Appendix S1.

**Fig. 2:**
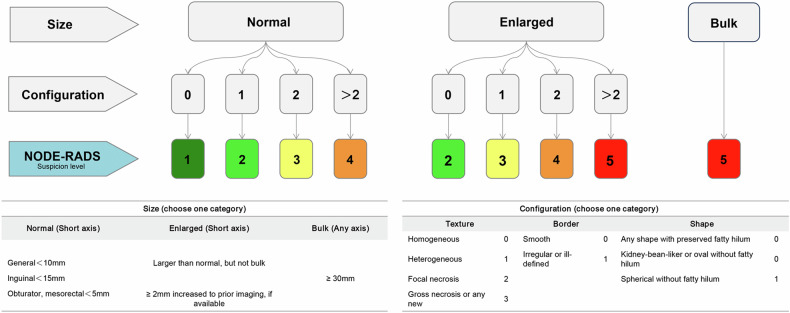
The Node RADS classification system was adapted from the original publication [18]

Radiological assessment was conducted independently by two readers (A and B), each possessing over 7 and 10 years of experience in genitourinary imaging. They followed the Node-RADS 1.0 criteria from 2021, blinded to histopathology results (Fig. 2). Regional lymph nodes were categorized into three types: Hilar, Side-specific (pre-/para-aortic or pre-/para-caval), and Inter-aorto-caval.

**Fig. 3:**
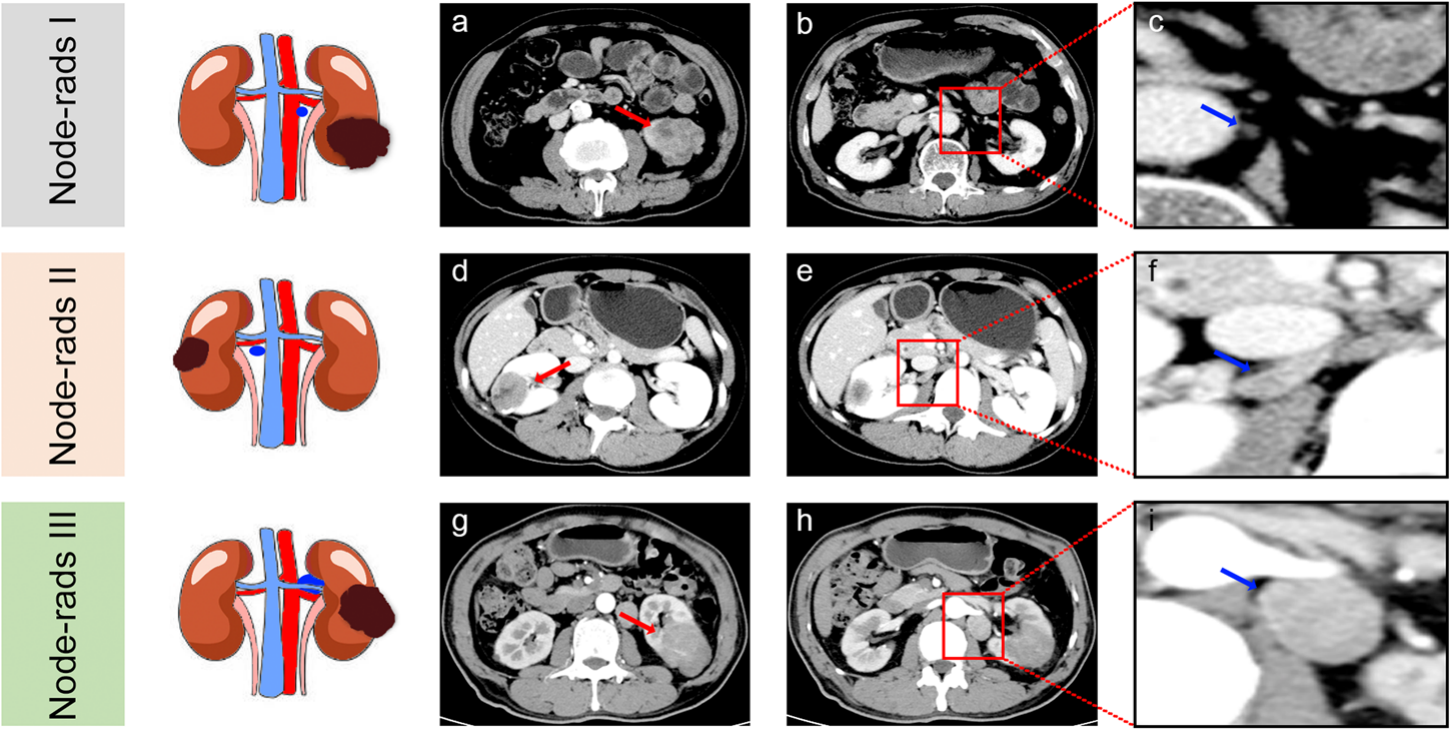
Representative example of Node-RADS scores. The red arrow indicates the primary tumor, the red box indicates the selected regional lymph node and the blue arrow indicates the magnified selected lymph node. **a**–**c** A 30-year-old male with pathological stage T3a and tumor size 4.6 cm showed no recurrence, progression, or death during the 161.3 months of follow-up. The selected lymph nodes, measuring 9 × 6 mm, exhibited uniform texture, smooth borders, and a kidney bean shape, resulting in a lymph node score of 1 point (Node-rads I). **d**–**f** A 23-year-old female with pathological stage T1a and tumor size 3.4 cm experienced progression during the 57 months of follow-up but is still alive. The lymph nodes in this case, measuring 16 × 6 mm, were heterogeneous with smooth borders and a kidney bean shape, leading to a lymph node score of 3 points (Node-rads II). **g**–**i** A 51-year-old man with pathological stage T1b and tumor size 5.2 cm faced progression and death during the 21 months of follow-up. The enlarged lymph node, measuring 27 × 17 mm, was heterogeneous, smooth in border, and spherical in shape, resulting in a lymph node score of 4 points (Node-rads III)

Nodes were evaluated using the Node-RADS system on CT scans, which employs a three-level flowchart (see Fig. 2) to classify nodes based on size and configuration. Each node is assigned a score from 1 to 5, indicating the suspicion level for malignancy: “1—very low”; “2—low”; “3—equivocal”; “4—high”; “5—very high”. To simplify, scores 1 and 2 are categorized as Node-RADS I, score 3 as Node-RADS II, and scores 4 and 5 as Node-RADS III (Fig. 3). Details on how the two radiologists performed the assessment were provided in Appendix S2. Interreader and intrareader agreement of both radiologists was calculated.

### Statistical analysis

We conducted statistical analyses to compare baseline characteristics and clinicopathological features among the three Node-RADS categories of pRCC. Continuous variables were compared using the Kruskal–Wallis test. Categorical variables were evaluated using either the chi-square test or Fisher’s exact test. Kaplan–Meier method with log-rank test was used to calculate and compare progression-free survival (PFS) and cancer-specific survival (CSS) rates. Univariate and multivariate Cox regression analyses were performed to identify independent predictors. Variables demonstrating *p*-values < 0.05 in the univariate analysis were included in the multivariate analysis, which employed backward stepwise selection based on the Akaike Information Criterion (AIC). Inter- and intra-observer agreement for the Node-RADS scores and categories of pRCC were assessed using Cohen weighted kappa. The detailed pRCC risk stratification by Leibovich et al [26] is shown in Appendix S3. To assess the incremental value of the Node-RADS category within the Leibovich model for survival prediction, we constructed a fusion model that integrates the Node-RADS category with the Leibovich group in a multivariate analysis. Subsequently, we compared the performance of the fusion model that incorporates the Node-RADS category and Leibovich group against the original Leibovich model. The prognostic performance of the model was evaluated using the concordance index (C-index) and time-dependent area under the curve (time-AUC) of receiver operating characteristic (ROC) curves. Internal validation was performed using 1000 bootstrap samples, presenting C-index. Statistical analysis was carried out utilizing SPSS v.20.0 and R software (version 4.2.0). A significance level of *p* < 0.05 was applied.

## Results

### Patients characterization

Following the selection procedure, a total of 218 patients were enrolled in our study (Fig. 1). The median follow-up was 57.3 months (IQR: 30.05–88.72) for progression-free survival and 59.15 months (IQR: 34.25–89.45) for cancer-specific survival at the last follow-up. Twenty-five (11.5%) patients experienced progression, and 15 (6.9%) patients died from pRCC. The identified sites of distant metastasis included the liver (*n* = 9), lung (*n* = 11), lymph nodes (*n *= 10), bone (*n* = 6), and brain (*n* = 1). Additionally, local recurrence (*n* = 2) was found in the local ipsilateral region. The observed progression-free survival rates were 92.20% at 3 years, 89.45% at 5 years, and 88.99% at 10 years, while cancer-specific survival rates were 96.33% at 3 years, 94.04% at 5 years, and 93.12% at 10 years (Table S1). Patient characteristics are summarized in Table 1. Among the 218 cases of pRCC, only 8 (3.7%) underwent lymph node dissection (LND), revealing 7 (3.2%) with pN1M0 pRCC. Additionally, 186 (85.3%), 10 (4.6%), and 22 (10.1%) cases were classified as Node-rads I, Node-rads II, and Node-rads III, respectively (Tables 2 and 3). Among these Node-rads III patients, 16 out of 22 (72.73%) patients experienced disease progression, including 1 had local recurrence and 15 developed distant metastases. Additionally, 11 out of 22 (50%) Node-rads III patients experienced cancer-related mortality (Table S1).

**Table 1:** Patient characteristics

Feature	pRCC (*N* = 218)
Median age at surgery, year (IQR)	58 (52–65)
Male sex (%)	164 (75)
Symptom (%)	46 (21)
Surgery type (%)	
Radical/partial	97 (44)/121 (56)
Procedure type (%)	
Open/laparoscopic	99 (45)/119 (55)
Lymphadenectomy (%)	7 (3)
ECOG-PS (%)	
0/1–4	174 (80)/44 (20)
Adjuvant treatment	
Yes/No	14 (6)/204 (94)
Subtype (%)	
Type I/II/unclassified	73 (33)/91 (42)/54 (25)
2010 pT stage (%)	
pT1a	115 (53)
pT1b	60 (27)
pT2a	14 (6)
pT2b	3 (1)
pT3a	14 (6)
pT3b	11 (5)
pT3c	0
pT4	1 (< 1)
Median tumor size, cm (IQR)	3.5 (2.5–5.3)
WHO/ISUP grade (%)	
1/2/3/4	10 (5)/147 (67)/57 (26)/4 (2)
Sarcomatoid differentiation (%)	3 (1)
Pernephric or renal sinus fat invasion (%)	22 (10)
Tumor thrombus present (%)	12 (6)
Tumor necrosis (%)	60 (28)
Nodal involvement (2010 pN- classification) (%)	
pN_0/x_ /pN_1_	211 (97)/7 (3)

*IQR* interquartile range, *pRCC* papillary renal cell carcinoma, *ECOG-PS* Eastern Cooperative Oncology Group Performance Status, *WHO/ISUP* World Health Organization/International Society of Urological Pathology

**Table 2:** Three-tier classification of Node-RADS scores

Node-RADS score subdivision			
Node-RADS categories	I	II	III
Node-RADS score	1–2	3	4–5

*Node-RADS* Node Reporting and Data System

**Table 3:** Clinicopathological features among different CT-based Node-RADS categories

Variable	Node-rads I (*n* = 186)	Node-rads II (*n* = 10)	Node-rads III (*n* = 22)	*p*-value*
Tumor size	3.50 [2.50; 4.80]	4.10 [2.97; 5.97]	5.35 [3.17; 8.82]	0.007
Subtype				0.261
Type I	66 (35.48%)	4 (40%)	3 (13.64%)	
Type II	74 (39.78%)	4 (40%)	13 (59.09%)	
Unclassified	46 (24.73%)	2 (20%)	6 (27.27%)	
WHO/ISUP grade				0.001
Low-grade	141 (75.81%)	8 (80%)	8 (36.36%)	
High-grade	45 (24.19%)	2 (20%)	14 (63.64%)	
Tumor necrosis				0.540
Absent	137 (73.58%)	7 (70%)	14 (63.64%)	
Present	49 (26.42%)	3 (30%)	8 (36.36%)	
Sarcomatoid differentiation				0.380
Absent	184 (98.92%)	10 (100.00%)	21 (95.45%)	
Present	2 (1.08%)	0 (0.00%)	1 (4.55%)	
Pernephric or renal sinus fat invasion				< 0.001
Absent	175 (97.85%)	7 (70%)	14 (63.64%)	
Present	11 (5.91%)	3 (30%)	8 (36.36%)	
Tumor thrombus				< 0.001
Absent	182 (97.93%)	10 (100.00%)	14 (63.64%)	
Present	4 (2.07%)	0 (0.00%)	8 (36.36%)	
Nodal involvement (2010 pN-classification)				< 0.001
pN0/x	185 (99.46%)	10 (100%)	16 (72.73%)	
pN1	1 (0.54%)	0 (0%)	6 (27.27%)	

*Node-RADS* Node Reporting and Data System, *WHO/ISUP* World Health Organization/International Society of Urological Pathology

Categorical variables are expressed as numbers of patients, with percentages in parentheses, and were compared using Pearson’s χ^2^ test or Fisher exact test. Continuous variables are expressed as medians, with IQRs in parentheses, and were compared using the Kruskal–Wallis test

* The adjusted *p*-values after applying the False Discovery Rate (Benjamini and Hochberg) method

### Correlation between clinicopathological characteristics and Node-RADS categories of pRCC

Table 3 contains the correlation analysis of clinicopathological characteristics and three categories of lymph Node-RADS. The three Node-Rads categories differed in terms of tumor size (*p* = 0.007), WHO/ISUP grade (*p* = 0.001), perirenal or renal sinus fat invasion (*p* < 0.001), tumor thrombus (*p* < 0.001), and pathological lymph node involvement (*p* < 0.001). Two radiologists independently assessed Node-RADS scores and categories on preoperative CT, showing excellent interobserver and intra-observer agreement (κ = 0.951–0.988, all *p* < 0.001) (Table S2).

### Survival outcomes

#### Correlation between the Node-RADS categories and survival outcomes in pRCC patients

**Fig. 4:**
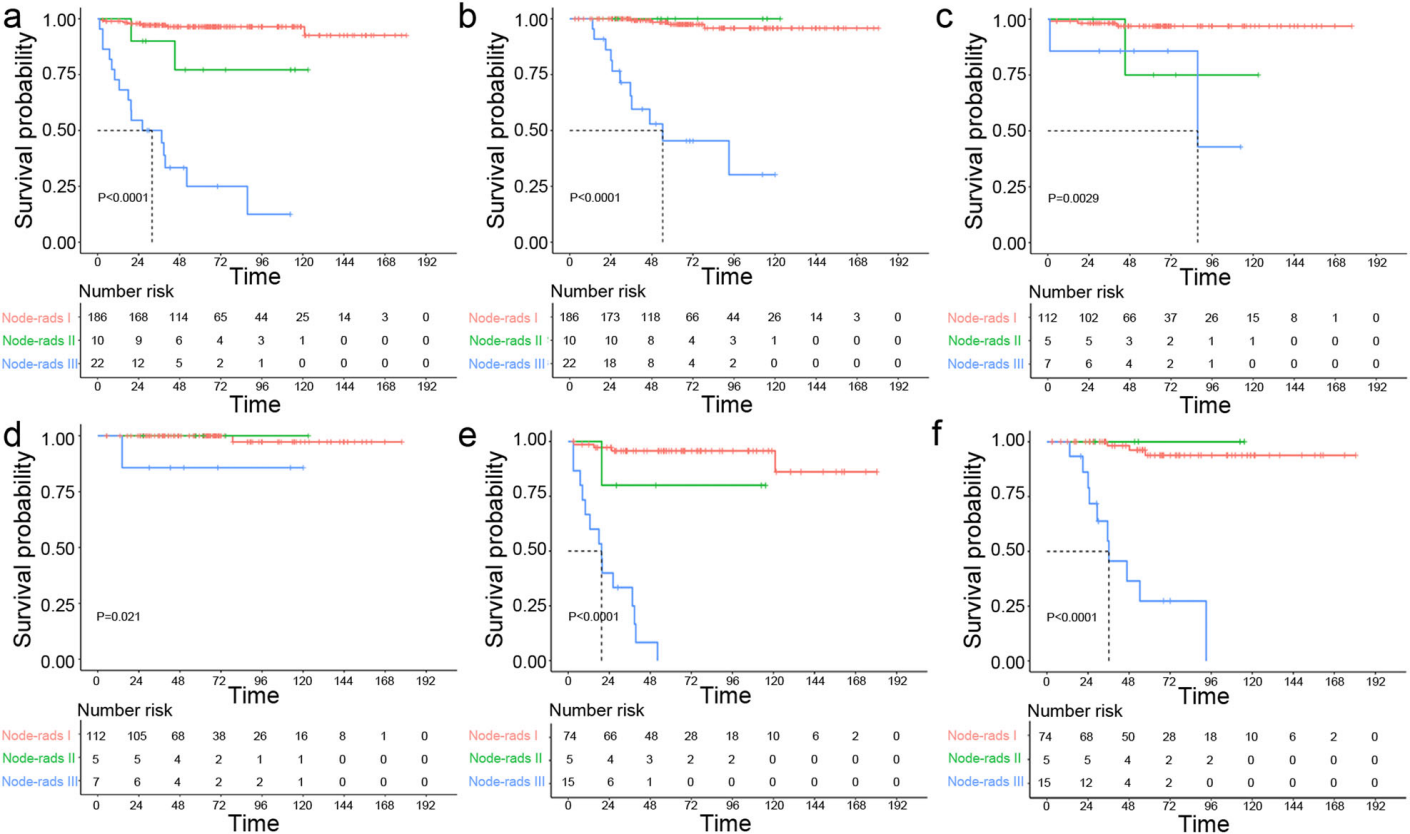
Kaplan–Meier curves for progression-free survival (PFS) and cancer-specific survival (CSS); comparison of PFS (**a**) and CSS (**b**) curves of patients with Node-RADS categories of pRCC; subgroup analysis of PFS (**c**) and CSS (**d**) of patients with tumors smaller than 4 cm and PFS (**e**) and CSS (**f**) of patients with tumors larger than 4 cm

The comparison of survival rates among the three categories of pRCC is presented in Table S1. Postoperative PFS rates were significantly lower among Node-rads III and Node-rads II pRCC patients compared to Node-rads I (50% versus 90% versus 97.3% at 3 years, 31.82% versus 80% versus 96.77% at 5 years, and 27.27% versus 80% versus 96.77% at 10 years, *p* < 0.001). Similarly, the cancer-specific survival rate of patients with Node-rads III pRCC was significantly lower than that of patients with Node-rads II and Node-rads I pRCC (68.18% versus 100% versus 99.46% at 3 years, 54.55% versus 100% versus 98.39% at 5 years, and 50% versus 100% versus 97.85% at 10 years, *p* < 0.001). Postoperative progression-free survival (PFS) and cancer-specific survival (CSS) curves for patients with different categories are depicted in Fig. 4a and Fig. 4b, respectively. As shown in Fig. 4c and Fig. 4d, there were significant differences in PFS and CSS among the three categories in patients with tumors smaller than 4 cm. Likewise, in the subgroup of patients with tumors larger than 4 cm, there were significant differences in PFS and CSS between the three categories (Fig. 4e, f).

#### Node-RADS Categories—an independent parameter for predicting PFS and CSS of pRCC

Table 4 displays the findings of the Cox regression analysis on progression-free survival (PFS) and cancer-specific survival (CSS). After multivariate analysis, Node-RADS (III vs II vs I, HR = 4.425, 95% CI = 2.494–7.851, *p* < 0.001), adjuvant treatment (HR = 3.24, 95% CI = 1.178–8.911, *p* = 0.023), and type of surgery (HR = 3.926, 95% CI = 1.43–10.77, *p* = 0.008) emerged as significant independent prognostic factors for PFS. Meanwhile, Node-RADS (III vs II vs I, HR = 4.466, 95% CI = 2.219–8.98, *p* < 0.001) and the presence of tumor thrombus (HR = 5.307, 95% CI = 1.047–26.88, *p* = 0.044) were identified as key prognostic factors for CSS on multivariable analysis based on Akaike information criterion (AIC) (Table 4 and Supplementary Table S3). After adjusting for clinicopathological variables, the Node-RADS category remained an independent prognostic factor for predicting both PFS and CSS (all *p* < 0.001, Supplementary Table S4).

**Table 4:** Univariable and multivariable Cox regression analyses for progression-free survival and cancer-specific survival in patients with papillary renal cell carcinoma

	Progression-free survival				Cancer-specific survival			
Feature	UV		MV		UV		MV	
	HR (95% CI)	*p*-value	HR (95% CI)	*p*-value	HR (95% CI)	*p*-value	HR (95% CI)	*p*-value
Age at surgery	0.977 (0.946–1.009)	0.155			0.975 (0.935–1.016)	0.233		
Sex (man as ref.)	0.556 (0.245–1.262)	0.160			0.673 (0.23–1.974)	0.471		
Symptom (absent as ref.)	4.355 (1.983–9.562)	< 0.001			7.235 (2.472–21.17)	< 0.001		
Surgery type (partial as ref.)	4.936 (1.848–13.18)	0.001	3.926 (1.43–10.77)	0.008	65596 (0-inf)	0.997		
Procedure type (laparoscopic as ref.)	1.146 (0.521–2.521)	0.734			1.414 (0.5–3.999)	0.514		
Lymphadenectomy (absent as ref.)	12.27 (4.534–33.2)	< 0.001			10.67 (2.988–38.15)	< 0.001		
ECOG-PS (0 as ref.)	1.409 (0.587–3.381)	0.443			1.228 (0.389–3.876)	0.726		
Adjuvant treatment (absent as ref.)	27.24 (11.61–63.91)	< 0.001	3.24 (1.178–8.911)	0.023	25.86 (9.265–72.18)	< 0.001		
Subtype (mixed as ref.)								
Type I	0.471 (0.105–2.105)	0.324			0.206 (0.021–1.98)	0.171		
Type II	2.628 (0.889–7.769)	0.081			2.107 (0.588–7.558)	0.253		
Tumor size	1.316 (1.183–1.462)	< 0.001			1.38 (1.218–1.563)	< 0.001		
ISUP grade (low-grade as ref.)	4.491 (2.013–10.01)	< 0.001			8.809 (2.8–27.71)	< 0.001		
Sarcomatoid differentiation (absent as ref.)	4.734 (0.636–35.21)	0.129			10.85 (1.402–84.10)	0.022		
Pernephric or renal sinus fat invasion (absent as ref.)	9.742 (4.39–21.61)	< 0.001			12.67 (4.586–35.02)	< 0.001	3.288 (0.732–14.76)	0.12
Tumor thrombus present (absent as ref.)	13.86 (5.934–32.41)	< 0.001			26.45 (9.509–73.60)	< 0.001	5.307 (1.047–26.88)	0.044
Tumor necrosis (absent as ref.)	1.719 (0.771–3.833)	0.185			1.248 (0.425–3.662)	0.687		
Nodal involvement (absent as ref.)	39.37 (14.49–106.9)	< 0.001			22.17 (7.488–65.66)	< 0.001		
Node-RADS (III vs II vs I)	5.879 (3.69–9.367)	< 0.001	4.425 (2.494–7.851)	< 0.001	6.118 (3.332–11.23)	< 0.001	4.466 (2.219–8.98)	< 0.001

*UV* univariable Cox regression analyses, *MV* multivariable Cox regression analyses, *HR* hazard ratio, *ECOG-PS* Eastern Cooperative Oncology Group Performance Status, *WHO/ISUP* World Health Organization/International Society of Urological Pathology, *Node-RADS* Node Reporting and Data System

#### Comparison of model prediction performance

Using time-dependent AUC analysis, the combined model incorporating the Node-RADS category and Leibovich’s model demonstrated higher AUC values compared to either Leibovich’s model or Node-RADS alone for both progression-free survival (PFS) and cancer-specific survival (CSS) (Table 5 and Fig. 5). The combined model showed superior predictive performance for PFS and CSS compared to Leibovich’s pRCC model after 1000 bootstrap samples (C-index: 0.865 vs 0.755, *p* = 0.005 for PFS; 0.921 vs 0.835, *p* = 0.014 for CSS). Node-RADS exhibited significantly higher AUC values for PFS and CSS compared to the model based on lymph node status at 3-year (0.813 vs 0.672, *p* = 0.05), 5-year (0.850 vs 0.643, *p* < 0.001), and 10-year (0.882 vs 0.627, *p* < 0.001) time points (Table 5 and Fig. 5). Furthermore, Node-RADS also demonstrated significantly higher C-index values for PFS and CSS compared to lymph node status after 1000 bootstrap samples for internal validation (C-index: 0.819 vs 0.651, *p* < 0.001 for PFS; 0.855 vs 0.652, *p* < 0.001 for CSS).

**Table 5:** Comparison of model prediction performance

	PFS							CSS					
	3-yC	*p*-value	5-yC		*p*-value	10-yC	*p*-value	3-yC	*p*-value	5-yC	*p*-value	10-yC	*p*-value
NRL-model	0.864 (0.75–0.979)	-	0.9 (0.818–0.982)			0.915 (0.834–0.995)		0.888 (0.719–1.057)	-	0.921 (0.823–1.02)		0.947 (0.872–1.022)	
L-model	0.787 (0.66–0.914)	0.09^#^	0.757 (0.645–0.87)	0.003^#^		0.732 (0.604–0.86)	< 0.001^#^	0.77 (0.581–0.958)	0.10^#^	0.841 (0.721–0.96)	0.09^#^	0.761 (0.581–0.941)	0.04^#^
NR-model	0.813 (0.698–0.927)	0.18^#^	0.85 (0.756–0.949)	0.17^#^		0.882 (0.796–0.968)	0.36^#^	0.889 (0.751–1.027)	0.961^#^	0.828 (0.69–0.996)	0.09^#^	0.83 (0.688–0.972)	0.07^#^
LNS-model	0.672 (0.558–0.785)	0.055*	0.643 (0.551–0.735)	< 0.001*		0.627 (0.541–0.713)	< 0.001*	0.604 (0.46–0.749)	0.001*	0.642 (0.518–0.767)	< 0.001*	0.684 (0.542–0.825)	0.015*

*NRL-model* Node-RADS combined with Leibovich’s model, *L-model* Leibovich’s model, *NR-model* Node-RADS model, *LNS-model* lymph node status model, *3-yC* 3-year AUC, *5-yC* 5-year AUC, *10-yC* 10-year AUC, *PFS* progression-free survival, *CSS* cancer-specific survival

* The *p*-value is based on the comparison between NR-model and LNS-model for AUC

# The *p*-value is based on the comparison between NRL-model and two other models for AUC

**Fig. 5:**
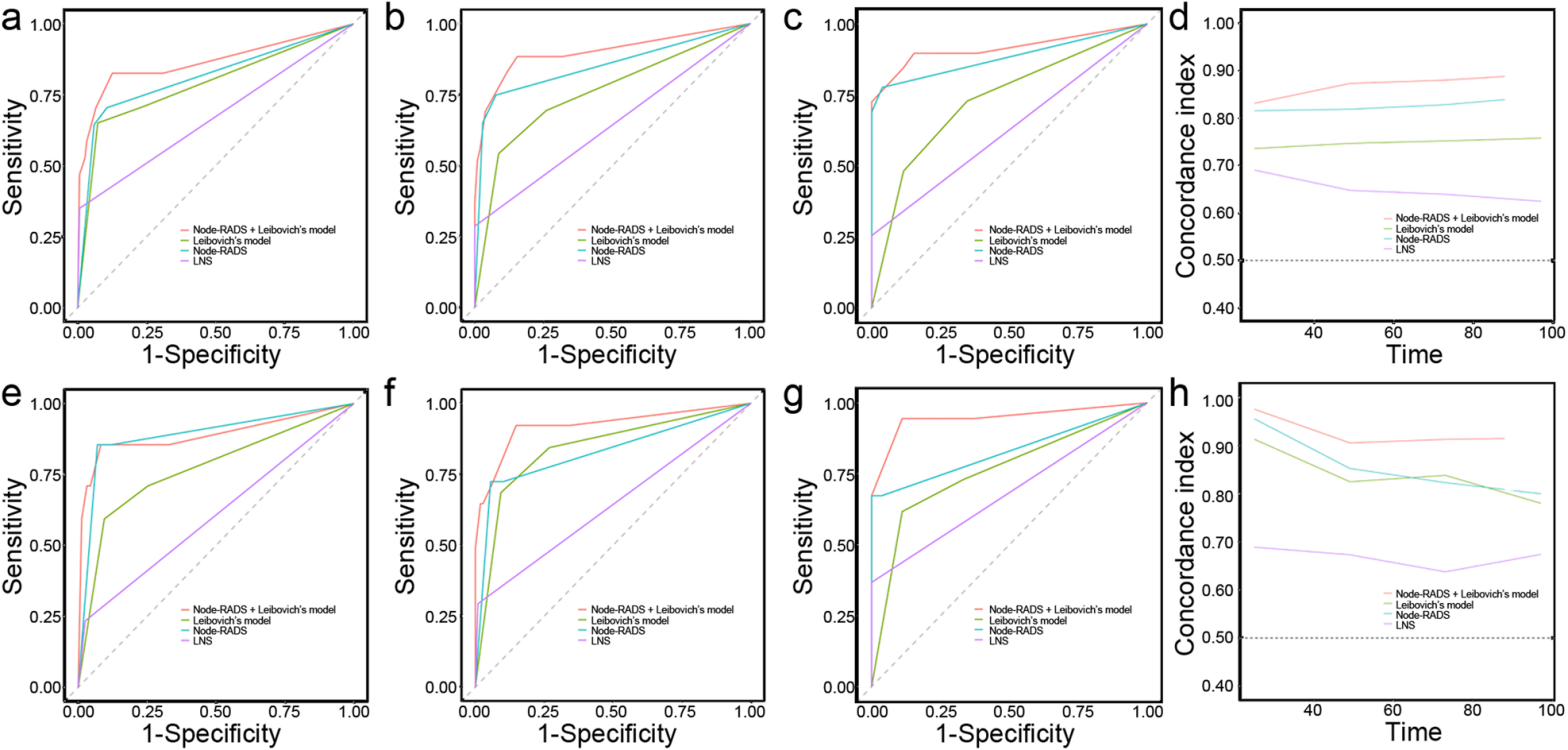
Graphs show discriminative performances. Time-dependent ROC curves for **a** 3-year, **b** 5-year, **c** 10-year and **d** time-dependent areas under the receiver operating characteristic curve measured model discriminatory performance at different points for PFS prediction. Time-dependent ROC curves for **e** 3-year, **f** 5-year, **g** 10-year and **h** time-dependent areas under the receiver operating characteristic curve measured model discriminatory performance at different points for CSS prediction

## Discussion

The preoperative imaging assessment of lymph node status is crucial for determining the prognosis in patients with papillary renal cell carcinoma (pRCC). Nonetheless, there is still no consensus on the optimal methods for radiological evaluation. In our study, we explored the implementation of a standardized Node Reporting and Data System (Node-RADS) for preoperative CT scans to assess the prognostic value of lymph nodes in pRCC. Our study showed that tumors classified with higher Node-RADS category (III vs II vs I) were significantly associated with poorer progression-free survival (PFS) and cancer-specific survival (CSS). In multivariable Cox regression analysis, Node-RADS category emerged as an independent predictor for both PFS and CSS, even after accounting for pathological node involvement. Additionally, our combined model integrating Node-RADS outperformed the Leibovich model alone in stratifying risk for pRCC within our cohort.

Recent studies have indicated that patients with nonmetastatic renal cell carcinoma (RCC) and lymph node involvement (LNI) often experience survival outcomes similar to those with metastatic RCC, highlighting the typically unfavorable prognosis associated with LNI [27, 28]. In response to this finding, earlier studies have utilized preoperative imaging to identify enlarged lymph nodes (≥ 1 cm) as a method to stratify RCC based on prognosis [16, 29]. Our findings suggest that the Node-RADS category, which integrates both size and configuration features into a standardized system for lymph node evaluation, can serve as a prognostic imaging biomarker for pRCC. Our study revealed a correlation between Node-RADS score and LNI, indicating that higher Node-RADS scores were closely associated with LNI and aggressive pathological characteristics such as larger tumor size, higher pathological grade, fat invasion, and venous tumor thrombus (all *p* < 0.001). These findings align with prior research investigating the association between lymph node involvement (LNI) and pathological characteristics [16, 30, 31]. In multivariable Cox analysis, the Node-RADS category emerged as an independent factor associated with both PFS and CSS, independent of pathologic node involvement. Moreover, the predictive accuracy of the model based on Node-RADS categories outperformed the model based on lymph node status (pN_1_ vs pN_0/X_) in predicting PFS and CSS.

The superior predictive performance of Node-RADS categories over lymph node status results can be attributed to several factors. One significant reason is the potential underestimation of pathological lymph nodes in clinical practice. It is crucial to note that post-surgery, only about 1% of patients with RCC are classified as LN + /M0 [9]. In our multicenter cohort, this percentage was approximately 3%. Moreover, the impact of pathologic nodal staging is difficult to evaluate due to the variability in lymph node dissection (LND) practices among RCC patients. The utility of LND in nonmetastatic RCC patients remains contentious due to conflicting evidence [11], leading to an observational bias where only selected patients undergo LND and can be identified as pathologically node-positive. Even among those undergoing LND, there exists a selection bias owing to the lack of standardized criteria for the extent of LND [12]. Given these challenges, preoperative imaging evaluation of lymph nodes assumes a critical role in identifying high-risk patients and tailoring personalized treatment strategies. For instance, in scenarios where high-risk patients might be overlooked for lymph node dissection or where positive lymph nodes could be missed due to non-standardized dissection protocols, preoperative imaging based on the RADS score can aid in identifying high-risk patients for more intensive monitoring or inclusion in adjuvant therapy trials, or in deciding on LND.

On the contrary, imaging demonstrates superior prognostic performance compared to pathology, primarily due to Node-RADS’ enhanced ability to quantify lymph nodes, thereby increasing their prognostic significance. Node-RADS evaluates not only lymph node size but also texture, improving the detection of micrometastatic nodes measuring less than 10 mm. Conversely, inflammatory enlargement of lymph nodes may influence kidney cancer prognosis. Studies indicate that clinical lymphadenopathy (≥ 1 cm) is common in patients with renal cell carcinoma and larger tumors, yet less than half of these cases show pathological lymph node involvement [32]. This phenomenon may relate to the inflammatory response and its impact on oncological outcomes [33]. Furthermore, some studies suggest that inflammatory lymphadenopathy (cN1pN0) could indicate more aggressive disease [29]. Due to the low rate of lymph node dissection (LND) in our cohort, we were unable to explore the relationship between Node-RADS, lymph node involvement (LNI), and inflammatory lymphadenopathy thoroughly. Given the conflicting evidence, accurately assessing lymph node risk and reaffirming their critical role is essential for selecting patients who would benefit most from LND and tailored postoperative treatment. Current guidelines advocate LND solely for renal cell carcinoma patients with clinically suspicious lymph nodes on imaging [31, 34, 35], potentially refining risk stratification and aiding in the selection of high-risk patients for further intervention.

In our multivariable Cox analysis, we identified adjuvant therapy (HR, 3.24) and radical nephrectomy (HR, 3.926) as independent predictors of recurrence risk. This aligns with previous studies suggesting that partial nephrectomy leads to lower recurrence rates compared to radical nephrectomy [36]. Adjuvant therapies are typically recommended for patients with advanced disease or higher risk profiles. Therefore, it is possible that adjuvant therapy is a response to the patient’s high-risk status rather than a direct cause of increased risk. Our findings suggest that Node-RADS and venous thrombosis independently contribute to prognostic value for Cancer-Specific Survival (CSS). Although factors like fat invasion and grade are significant, their impact may be overshadowed by the strong correlation with Node-RADS, potentially due to collinearity effects.

Our study had several limitations. First, it was retrospective, which could have introduced sample selection bias and resulted in an imbalanced dataset. Although our study sample is relatively unbalanced, the statistical analyses concerning the power of the study (see Appendix S4, Table S5) suggest that our results are reliable. Our current sample represents the largest collection of imaging data available, and we agree that these findings should be further prospectively validated by other researchers. Second, despite being based on multicenter data, the sample size remained small. Third, the low rate (3%) of lymph node dissection (LND) in our cohort resulted in only 7 patients with pathologically positive lymph nodes, limiting the evaluation of Node-RADS’ diagnostic performance and potentially underestimating the risk of lymph node involvement (LNI). Additionally, while the study considered lymph node size and anatomical location, there was uncertainty regarding whether the pathologically analyzed lymph node corresponded to the one expected based on imaging, as the same time, smaller lymph nodes posed challenges in assessing their imaging characteristics. Moreover, the World Health Organization (WHO) classification 2022 eliminated to classify papillary renal cell carcinoma (PRCC) into Type I and Type II. However, due to the limitations inherent in our retrospective study, the majority of cases were collected prior to 2022 so we continued to utilize the 2017 WHO classification.

## Conclusions

In summary, our study highlights the valuable prognostic information provided by preoperative Node-RADS in pRCC, an aspect that has been previously overlooked and underestimated. Node-RADS demonstrates practicality and reliability in predicting PFS and CSS among patients diagnosed with pRCC. Given the retrospective nature of our study, we advocate for prospective randomized controlled trials to further validate these findings in a larger cohort.

## Supplementary information

ELECTRONIC SUPPLEMENTARY MATERIAL

## Abbreviations

CSS: Cancer-specific survival
CT: Computed tomography
ECOG-PS: Eastern Cooperative Oncology Group Performance Status
HR: Hazard ratio
IQR: Interquartile range
LND: Lymph node dissection
LNI: Lymph node involvement
Node-RADS: Node Reporting and Data System
PFS: Progression-free survival
pRCC: Papillary renal cell carcinoma
