# Supplementary material for: Node-RADS category on preoperative CT predicts prognosis in patients with papillary renal cell carcinoma
Source: Eur Radiol. 2025 Feb 20;35(9):5480–91. doi: 10.1007/s00330-025-11446-y (PMC12350559; doi:10.1007/s00330-025-11446-y)
Supplement: Supplementary file 1 — ELECTRONIC SUPPLEMENTARY MATERIAL [file 330_2025_11446_MOESM1_ESM.pdf]

Node-RADS category on preoperative CT predicts prognosis in patients with papillary renal cell carcinoma

ELECTRONIC SUPPLEMENTARY MATERIAL

Appendix S1

Our hospital uses the following multidetector CT scanners: GE Lightspeed 64 (Ge Healthcare Chicago, Il, USA), Siemens Sensation 16 (Siemens Healthineers, Erlangen, Germany) and Siemens Definition 64 (Siemens Healthineers). The imaging parameters included a slice thickness/gap of 5 mm with a 5 mm interval and a tube voltage of 120 kVp. Corticomedullary and nephrographic phase images were captured at 20-40 seconds and 80-90 seconds post-injection of contrast material, respectively.

Appendix S2

Two radiologists initially selected the largest regional lymph node for evaluation. To ensure consistency in assessing the same lymph node, a specialized radiologist conducted a review in cases of uncertainty. If the initial evaluations differed by two or more scores (for example, if one radiologist assigned a score of 2 while the other assigned a score of 4), the specialized radiologist re-evaluated the case to confirm that the same lymph node was being assessed. Subsequently, both radiologists rescored the case. Radiologist A assessed the images twice, with a three-month interval between assessments, referred to as A1 and B. During each assessment, both radiologists independently evaluated and scored all 218 patients.

Appendix S3

Leibovich et al. [1] was conducted to validate using central data. This involved stratifying patients into group 1 (grades 1–2 without fat invasion and thrombus), group 2 (grade 3 without fat invasion and thrombus), and group 3 (grade 4 or

with fat invasion or any thrombus level) according to Leibovich's model.

Reference

(1) Leibovich BC, Lohse CM, Cheville JC et al (2018) Predicting Oncologic Outcomes in Renal Cell Carcinoma After Surgery. Eur Urol 73: 772-780.

Appendix S4

Using PASS software, we calculated the necessary sample sizes for the Cox proportional hazards regression [1]. The progression-free survival (PFS) model required N=114, while the cancer-specific survival (CSS) model required N=171 (as shown in the table S3). Our total sample size of 218 exceeds both of these requirements.

Reference

(1) Hsieh FY, Lavori PW. Sample-size calculations for the Cox proportional hazards regression model with nonbinary covariates. Control Clin Trials. 2000 Dec;21(6):552-60.

Table S1 Progression-free and cancer-specific survival outcomes of pRCC

Variables	Total (n=218)	Node-rads I (n=186)	Node-rads II (n=10)	Node-rads III (n=22)	P value
PFS, month	57.3 [30.05;88.72]	62.60 [33.83;90.30]	56.45 [32.38;103.30]	27.60 [10.68;41.60]	＜0.001
Relapse (%)	25(11.47)	7(3.76)	2(20)	16(72.73)	＜0.001
3-year PFS rate, %	92.20	97.31	90	50	＜0.001
5-year PFS rate, %	89.45	96.77	80	31.82	＜0.001
10-year PFS rate, %	88.99	96.77	80	27.27	＜0.001
CSS, month	59.15 [34.25;89.45]	63.05 [35.60;90.30]	59.50 [51.73;103.30]	35.85 [25.85;64.65]	0.032
Died of tumor (%)	15 (6.88)	4 (2.15)	0 (0)	11 (50)	＜0.001
3-year CSS rate, %	96.33	99.46	100	68.18	＜0.001
5-year CSS rate, %	94.04	98.39	100	54.55	＜0.001
10-year CSS rate, %	93.12	97.85	100	50	＜0.001

PFS, Progression-free survival; CSS, cancer-specific survival. Bold values show statistically significant differences.

Table S2 Kappa analysis of two radiologists on 218 patients.

	Frequency			K value (A1 and A2)	K value (A1 and B)	Agreement
	A1	A2	B			
Node-rads score				0.963 (0.955-0.971)	0.951 (0.941-0.961)	Excellent
1	152 (70)	163 (75)	168 (77)	…	…	…
2	34 (16)	25 (11)	21 (10)	…	…	…
3	10 (4)	10 (5)	8 (4)	…	…	…
4	10 (4)	8 (4)	9 (4)	…	…	…
5	12 (6)	12 (5)	12 (5)	…	…	…
Node-rads categories				0.988 (0.984-0.992)	0.987 (0.982-0.991)	Excellent
I	186 (85)	188 (86)	189 (87)	…	…	…
II	10 (5)	9 (4)	8 (3)	…	…	…
III	22 (10)	21 (10)	21 (10)	…	…	…

Interobserver and intraobserver agreement was assessed using Cohen weighted kappa statistics. A1 and A2 were measured twice by the first radiologist, who has 7 years of experience, while B was measured by a second radiologist with 10 years of experience. Agreement was considered poor (κ or ICC < 0.2), fair (κ or ICC: 0.2–0.4), moderate (κ or ICC: 0.4–0.6), substantial (κ or ICC: 0.6–0.8), or excellent (κ or ICC > 0.8).

Table S3 The VIF value of retained variables in the models.

PFS-model (AIC = 185.12)		CSS-model (AIC = 100.26)	
Factor	VIF	Factor	VIF
Surgery type	1.000	Pernephric or renal sinus fat invasion	1.542
Adjuvant treatment	1.385	Tumor thrombus present	1.630
Node-RADS	1.387	Node-RADS	1.107

VIF, variance inflation factor; AIC, Akaike Information Criterion; Node-RADS, Node Reporting and Data System.

Table S4 Multivariate Cox regression analysis of the Node-RADS categories with PFS and CSS

	PFS		CSS	
	HR (95%CI)	P value	HR (95%CI)	P value
Age at surgery	0.969 (0.922-1.019)	0.219	0.986 (0.942-1.033)	0.553
Sex (man as ref.)	0.442 (0.154-1.272)	0.13	1.052 (0.334-3.318)	0.931
Symptom (absent as ref.)	0.589 (0.131-2.646)	0.49	1.721 (0.561-5.284)	0.343
Surgery type (partial as ref.)	3.332 (0.924-12.01)	0.066	0.475(0-1.145)	0.965
Procedure type (laparoscopic as ref.)	1.137 (0.342-3.787)	0.834	4.369 (1.446-13.20)	0.009
Lymphadenectomy (absent as ref.)	0.856 (0.155-4.735)	0.858	3.058 (0.778-12.02)	0.11
ECOG-PS (0 as ref.)	0.696 (0.172-2.823)	0.612	0.348 (0.106-1.143)	0.082
Adjuvant treatment (absent as ref.)	1.753 (0.259-11.84)	0.565	0.35 (0.114-1.075)	0.067
Subtype (mixed as ref.)				
Type I	0.967 (0.167-5.587)	0.97	0.952 (0.12-7.581)	0.963
Type II	2.219 (0.563-8.75)	0.255	2.76 (0.832-9.162)	0.097
Tumor size	0.9 (0.714-1.135)	0.372	1.087 (0.925-1.276)	0.311
ISUP grade (low-grade as ref.)	1.083 (0.309-3.799)	0.901	1.289 (0.342-4.859)	0.707
Sarcomatoid differentiation (absent as ref.)	13.19 (0.444-392.0)	0.136	52.83 (4.876-572.4)	0.001

Pernephric or renal sinus fat invasion (absent as ref.)	3.466 (0.842-14.26)	0.085	3.833 (1.216-12.08)	0.022
Tumor thrombus present (absent as ref.)	0.876 (0.089-8.615)	0.91	3.836 (1.178-12.48)	0.026
Tumor necrosis (absent as ref.)	0.869 (0.274-2.755)	0.812	0.28 (0.092-0.852)	0.025
Nodal involvement (absent as ref.)	3.714 (0.46-29.99)	0.218	0.404 (0.128-1.278)	0.123
Node-RADS (III vs II vs I)	4.778 (2.545-8.969)	＜0.001	7.65 (3.838-15.25)	＜0.001

PFS, Progression-free survival; CSS, cancer-specific survival；HR, hazard ratio；ECOG-PS, Eastern Cooperative Oncology Group Performance Status; WHO/ISUP, the World Health Organization/International Society of Urological Pathology. Node-RADS, Node Reporting and Data System.

Table S5 The results of sample-size calculations for the cox proportional hazards regression model.

	PFS-model	CSS-model
Power	0.80	0.80
Alpha	0.05	0.05
P	0.1147	0.0688
B	1.487	1.496
R2	0.292	0.222
SD	0.62	0.62
Beta	0.199	0.198
N	114	171

Power is the probability of rejecting a false null hypothesis; Alpha is the probability of rejecting a true null hypothesis; P is the event rate; B is the size of the regression coefficent to be detected; R2 is the R-squared achieved when X1 is regressed on the other covariates; SD is the standard deviation of X1; Beta is the probability of accepting a false null hypothesis; N is the size of the sample drawn from the population.
